# Some Levels of Plasma Free Fatty Acids and Amino Acids in the Second Trimester Are Linked to Gestational Diabetes and Are Predictive of Persisting Impaired Glucose Tolerance After Delivery

**DOI:** 10.3390/jcm14134744

**Published:** 2025-07-04

**Authors:** Vendula Bartáková, Anna Pleskačová, Lukáš Pácal, Monika Skrutková Langmajerová, Jindra Smutná, Katarína Chalásová, Veronika Eclerová, Zdeněk Glatz, Kateřina Kaňková, Josef Tomandl

**Affiliations:** 1 Department of Pathophysiology, Faculty of Medicine, Masaryk University, Kamenice 5, 625 00 Brno, Czech Republic; pleskacova@mail.muni.cz (A.P.); paci@med.muni.cz (L.P.); katarina.kuricova@gmail.com (K.C.); kankov@med.muni.cz (K.K.); 2 Department of Biochemistry, Faculty of Medicine, Masaryk University, Kamenice 5, 625 00 Brno, Czech Republic; tomandl@med.muni.cz; 3 Department of Biochemistry, Faculty of Science, Masaryk University, Kamenice 5, 625 00 Brno, Czech Republic; monika.lang@gmail.com (M.S.L.); glatz@chemi.muni.cz (Z.G.); 4 Department of Mathematics and Statistics, Faculty of Science, Masaryk University, Kotlarska 2, 611 37 Brno, Czech Republic; eclerova@math.muni.cz

**Keywords:** gestational diabetes mellitus, amino acids, free fatty acids

## Abstract

**Background/Objectives:** Gestational diabetes mellitus (GDM) represents an increased metabolic risk in future life for both mother and child. We hypothesize free fatty acids (FFAs) and amino acids (AAs) disturbances in plasma during second trimester might be indicating high risk of persisting glucose intolerance (PGI). The aim of study was to determine plasma FFAs and AAs during pregnancy in women with normal pregnancy and GDM and also in post-GDM women with PGI after delivery and to find potential association of altered FFAs and AAs profile with adverse peripartal outcomes and PGI after GDM. **Material and Methods:** A total of 54 pregnant women were included in the study. Of those 34 participants had GDM. PGI was diagnosed by oGTT up to one year after delivery. Plasma FFAs were determined using GC-FID and plasma AAs levels were determined using CE-MS method. **Results:** Decreased levels of tetradecanoic acid and several AAs were found in GDM group during pregnancy. Oleic and docosahexaenoic acid correlated positively while almost all AAs negatively correlated with oGTT values in the pregnancy (all *p* < 0.05, Spearman). Logistic regression model (using AAs, FFAs and BMI) identified higher citrulline and glutamate levels and lower tetradecenoic acid and choline as the best predictors for postpartum PGI. Some differences in AA levels were detected in women with macrosomic babies. **Conclusions:** Data support a possible link between GDM development and PGI after delivery and selected metabolite levels. The predictive potential of plasma FFAs and AAs levels on a diabetes risk in future life requires further validation.

## 1. Introduction

Gestational diabetes mellitus (GDM) affects more than 15% of pregnancies in developed countries and represents the most common complication of pregnancy. GDM increases the risk of perinatal complications and adverse pregnancy outcomes. Furthermore, it also predisposes both mother and child to glucose intolerance or overt diabetes development later in life [1,2]. GDM usually manifests during the second trimester as a result of inadequate insulin production to match decreasing insulin sensitivity (caused by a combination of physiological mechanisms such as placental anti-insulin hormones and weight gain) and most likely pre-existing susceptibility (both genetic and lifestyle incl. age, overweight or marked obesity etc.) [3]. By definition, GDM should revert to normal glucose tolerance soon after delivery; however, a subset of women retains glucose abnormality postpartum, and it remains to be determined whether those are truly GDM subjects or women with type 2 diabetes mellitus (T2DM) first manifested in pregnancy. The latter represents the high-risk subgroup for early development of metabolic and cardiovascular consequences such as diabetic microvascular complications, atherosclerosis, cognitive decline etc. As shown recently by us, a substantial part of GDM subjects—around 22%—manifests three or more components of metabolic syndrome in the second trimester of pregnancy and this proportion further increases in those retaining postpartum glucose intolerance/diabetes (PGI) up to 40% [4]. This may suggest more complex effects and exposures of foetus/new-born to harmful metabolic signatures compared to GDM uncomplicated by metabolic syndrome.

Prevailing metabolic derangement in GDM (either with or without metabolic syndrome)—reduced insulin sensitivity with inappropriate insulin response—does not cause purely carbohydrate metabolism disturbances, but affects other nutrients too incl. lipid and protein metabolism. This might contribute to the altered nutrient turnover during pregnancy, affecting both maternal and foetal metabolism, as comprehensively reviewed recently [5]. Metabolomics has been used to analyse metabolic profiles and to identify novel biomarkers associated with insulin resistance and T2DM [5]. Since the pathophysiological mechanisms operating in T2DM might be, to some (or even a greater extent, in a subgroup of patients with metabolic syndrome) identical to those in GDM, the use of this approach might broaden our understanding of GDM. Furthermore, metabolomics may increase our ability to predict postpartum GDM/T2DM persistence and event. even quantify the risk of subsequent cardiovascular diseases among women and their offspring [6]. While several studies focused on amino acid (AA) and free fatty acid (FFA) metabolism in pregnancy complicated by GDM, the results of these studies are, unfortunately, inconsistent due to applying different diagnostic criteria for GDM historically and various sample collection periods. A brief summary of published data indicates significantly increased ornithine levels in women with GDM [7]. Rahimi et al. found higher arginine, glycine and methionine concentrations in GDM mothers [8]. Furthermore, increased alanine, glutamate and serine were found to have a predictive potential for GDM in the third trimester of pregnancy in a nested case-control study [9]. On the contrary, Pappa et al. found lower methionine, glycine, alanine, citrulline and ornithine levels in GDM subjects compared to controls [10]. One study comprising GDM and healthy pregnant controls focused on metabolic profile in pregnancy as well as postpartum [11] and showed GDM women having significantly increased postpartum levels of leucine, isoleucine and valine (11 ± 3 months after delivery) without significant differences between GDM and control group during pregnancy. Regarding FFAs, increased levels have already been observed in GDM [10,12,13]. The elevation of FFAs in pregnancy can contribute to the peripheral insulin resistance commonly observed during late pregnancy. High FFAs levels inhibit insulin-stimulated glucose uptake and oxidation and stimulate hepatic gluconeogenesis and also stimulate excessive insulin production. Placental FFAs transfer was previously thought as primarily gradient-dependent diffusion and thus GDM may result in an accelerated transfer into the foetus. It has been proposed that hyperinsulinemia and high FFAs are responsible for increased adipose-tissue mass of the foetus observed in GDM pregnancies [12]. However, recent data suggest that the placenta is dependent upon several fatty acid transport proteins for the uptake of fatty acids p-FABPpm, FATP and FAT/CD36 which are pivotal for the uptake of FFA. The concept of free diffusion has been shown to be incorrect [14]. CD36 in particular is important for LCFA uptake and that fits with the data presented, it also has strong links to the inflammatory process and again that fits with the observed difference in GDM vs. controls CD36 is increased in GDM especially in endothelial and parenchymal cells [15].

To our knowledge, no study explored AAs and/or FFAs as potential predictive biomarkers for peripartal adverse outcomes (both for mother and child) and persisting glucose intolerance after GDM in the indexed pregnancy. We hypothesize the disturbances in AAs and FFAs in the high-risk group of women (those with GDM, with any peripartal adverse outcomes or those with persisting postpartum glucose intolerance (PGI). Therefore, the aims of this study were (1) to determine mid-gestational fasting plasma levels of AAs and FFAs in pregnant women with or without GDM in Czech (solely Caucasian) population, (2) to find a potential disturbance pattern of FFAs and/or AAs associated with maternal and neonatal peripartal complications and adverse pregnancy outcomes and (3) to compare post-GDM women with persisting glucose intolerance (PGI) postpartum with those reverting to normal glucose tolerance as a way to identify potential biomarkers for the prediction of PGI after GDM.

## 2. Methods and Materials

### 2.1. Subjects

A total of 54 pregnant women were enrolled in the study (see Table 1). Both GDM cases (*n* = 34) and controls (*n* = 20 women with normal pregnancies) attended mid-gestational screening. This subgroup of women was part of a larger study of biomarkers predictive of PGI in GDM women described in detail in Bartakova et al., JCM, 2024 [4] and recent study subjects were participants whose aliquots of plasma samples in biobank were sufficicent to perform all analyses. Study was approved by the Ethical Committee of Faculty of Medicine, Masaryk University, Brno, Czech Republic, and was conducted in accordance with Helsinki declaration (approval number 22/2010, date of approval 16 September 2010). Each participant provided informed consent prior being included in the study.

GDM subjects also underwent postpartum oral glucose tolerance test (oGTT) 6 weeks to 12 months after delivery. Mid-gestational oGTT was carried out with 75 g glucose load between 24–28th weeks of pregnancy and the following thresholds were used to diagnose GDM with IADPSG criteria (FPG ≥ 5.1 mmol/L, 1-h post-load glucose ≥ 10.0 mmol/L and 2-h post-load glucose ≥ 8.5 mmol/L with any one of the three cut-off values qualifying for the GDM diagnosis). GDM women were followed from the time of GDM diagnosis till the birth at the Diabetes Centre of the University Hospital Brno where postpartum (6 weeks to 12 months after delivery) oGTT was carried out using standard WHO criteria for diabetes/prediabetes. The treatment for GDM included diet in all cases, 44.0% of GDM cases required insulin therapy. Exclusion criteria were diabetes before pregnancy, non-Caucasian origin and severe comorbidities.

Peripartal and biochemical parameters were retrieved from hospital electronic health records extracted by investigators, other data were obtained from questionnaires (available in authors) developed by investigators that were completed by study subjects and their diabetologist at the time of GDM diagnosis (second trimester of gravidity, after oGTT test). All data were transferred to the electronic database (available in authors). The following maternal parameters were available: age at the time of GDM diagnosis, glycaemia during oGTT test in the mid-trimester, family history of diabetes, history of previous GDM, pre-gestational BMI and weight gain during pregnancy. Analysed peripartal parameters comprised data on (i) length of delivery (≥480 min duration of all three stages starting with the first contraction and ending with placenta delivery of labour counted defined as prolonged), (ii) mode of delivery (need of delivery induction, instrumental delivery or Caesarean section), (iii) delivery complications (such as manual extraction of the placenta or uterine hypotonia) and, finally, (iv) selected neonatal parameters (Apgar score, pH of cord blood, base excess (BE) and child-birth weight). For details see Table 1 and Table 2. Samples of peripheral EDTA-blood were taken from each study participant during their scheduled visit in the prenatal or diabetes centre (approx. 1–2 weeks after GDM diagnosis/testing) at mid-trimester and repeatedly postpartum in the post-GDM group only. Plasma was separated by centrifugation (1000× *g*, 10 min, 4 °C) and stored at −70 °C until analysis.

### 2.2. Measurement of Plasma Free Fatty Acids

Following FFAs were measured: dodecanoic (C12:0), tetradecanoic (C14:0), palmitic (C16:0), stearic acid (C18:0), heptadecanoic acid (C17:0), tetradecenoic (C14:1), palmitooleic (C16:1), oleic (C18:1), linoleic (C18:2), linolenic (C18:3), eicosapentaenoic (C20:5) and docosahexaenoic acid (C22:6). The extraction and derivatization procedure was based on previously described method [16] with minor modifications. Given the limited initial volume of 180 µL of plasma only non-esterified fatty acid fraction was detected. All analyses were performed on Bruker 450-GC gas chromatography instrument equipped with a flame ionization detector (FID). A Stabilwax-MS (Restek, Bad Homburg, Germany) capillary column (fused silica, 30 m, 0.25 mm i.d., 0.25 µm df) was used.

### 2.3. Measurement of Plasma Amino Acids

A panel of 20 proteinogenic AAs (incl. all essential AAs), carnitine, acetyl-carnitine, choline, betaine, citrulline, ornithine, creatinine, collagen and hydroxyproline were measured in fasting plasma samples. The procedure of sample preparation was based on the organic precipitation of plasma proteins. An available aliquot of plasma (40 μL) was deproteinized by organic solvent and simultaneously internal standards were added (160 μL of 5 μmol L^−1^ butoxycarbonyl-arginine and 10 μmol L^−1^ methionine sulfone dissolved in acetonitrile:DMSO 80:20). Samples were thoroughly mixed, centrifuged at 12,100× *g* for 5 min and supernatants were used for analysis using capillary electrophoresis coupled with mass spectrometry (CE-MS). Agilent 7100 CE System (Agilent Technologies, Inc.; Santa Clara, CA, USA) was used for separation of analytes by bare fused silica capillary with i.d. 50 μm, length 95 cm, filled by 15% acetic acid and kept constantly at 35 °C. Rinsing of capillary before analysis was performed with following protocol: 50% acetic acid for 5 min, water for 1.5 min and 15% acetic acid for 5 min. The injection time was 40 s at 50 mbar and applied separation voltage was 30 kV in normal polarity. Capillary was flushed with 0.5 mL of water after analysis. CE was connected with MS detector Bruker quadrupole-time of flight Maxis via Agilent sheath-liquid interface equipped by Dionex UltiMate pump. Electrospray formation was supported by solution consisted of MeOH-water (1:1) with flow rate of 6 μL min^−1^. The nebulising pressure of nitrogen was set at 0.4 bar, the drying gas flow rate at 5 L min^−1^, the drying gas temperature at 250 °C and the electrospray voltage was set at 4.5 kV. Total analysis run time was 24 min. Quantification was based on external calibration curve. Cysteine was measured in oxidized dimeric form as cystine.

### 2.4. Statistical Analyses

Data were expressed as medians and interquartile ranges (IQR). Nonparametric tests were used for comparison between and within the groups (Mann-Whitney test and chi-square test). The area under the glucose curve (AUC_oGTT_) was computed from oGTT values using the trapezoid rule [17]. Correlations were computed using Pearson correlation coefficients. Software Statistica v14 (StatSoft, Tulsa, OK, USA) was used for all analyses. *p* < 0.05 was considered statistically significant.

Multivariate multinomial logistic regression model was created for measured amino acids and BMI with *p* < 0.1 for odds ratio (OR) for the univariate logistic regression model. For the final model, highly correlated variables were excluded. Five clusters of amino acids and BMI based on the Pearson correlation coefficient were created and only one variable from each cluster was used for further analyses. Multivariate multinomial logistic regression was performed, and we excluded variables with the highest *p* values. Finally, the multivariate multinomial logistic regression model was recomputed with the final set of variables. Multivariable analyses were conducted in R v4.4.0 (R Foundation for Statistical Computing, Vienna, Austria) using functions from the base stats package and the lmtest package for model inference, and the pROC package for ROC curve and AUC computation. Univariate and multivariate logistic models were constructed to determine an eventual statistically significant effect of any relevant variable and we used the pROC package in R to compute the ROC curve and AUC from model predictions, based on true class labels and fitted probabilities.

## 3. Results

### 3.1. Comparison of AAs and FFAs in GDM vs. Normal Pregnancy

**Figure 1:**
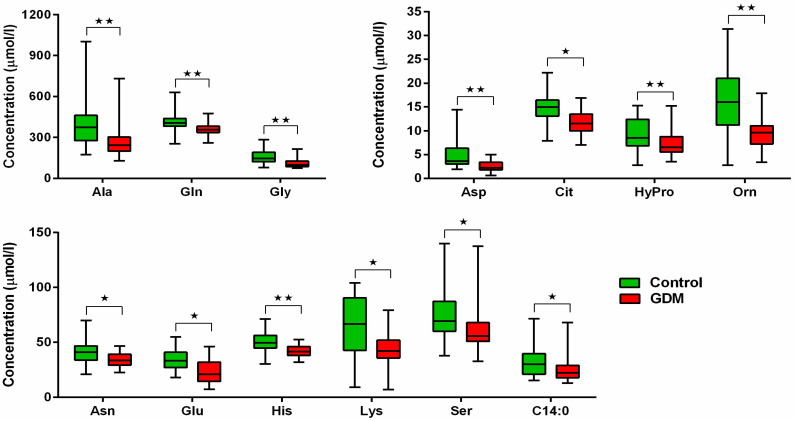
Comparison of FFAs and AAs levels during second trimester of pregnancy. ⋆ *p* < 0.05, ⋆⋆ *p* < 0.01.

Comparison of AAs revealed 12 (alanine, asparagine, asparagic acid, citrulline, glutamine, glutamic acid, glycine, histidine, hydroxyproline, lysine, ornithine and serine) out of 20 being decreased in GDM group in mid-gestation, compared to the control group, see Figure 1. Comparison of FFAs showed decreased levels of non-esterified tetradecanoic acid only in GDM women vs. controls (*p* = 0.03, Mann-Whitney), see Figure 1. There was no statistically significant difference between GDM and controls for the remaining FFAs. Moreover, no differences were found when the sum of all analysed FFAs and the sums of analysed saturated (SFAs), monosaturated (MUFAs) and polysaturaded (PUFAs) were calculated. Pairwise comparison within the GDM group showed that almost all AA levels increased postpartum compared to mid-gestation (all *p* < 0.05, Wilcoxon test), except for histidine and threonine. Conversely, levels of palmitooleic, heptadecanoic, oleic, linoleic, linolenic and docosahexaenoic acid within the GDM group significantly decreased postpartum (all *p* < 0.05, Wilcoxon test). Postpartum levels of docosahexaenoic acid were about one-third of their levels during pregnancy (*p* = 0.00009, Wilcoxon test). The sum of all analysed FFAs and also the sums of SFAs, MUFAs, and PUFAs were also significantly decreased postpartum in the GDM group (all *p* < 0.05, Wilcoxon test).

### 3.2. Correlation Between AAs and FFAs Levels and Biochemical and Anthropometric Parameters, Respectively

Regarding the AAs, FPG levels, 2-h PPG and AUC_oGTT_ were negatively correlated with glutamine, glycine, histidine, alanine, citrulline, glutamic acid, lysine, ornithine, serine and asparagine levels in the second trimester of pregnancy. Choline and valine levels were correlated positively with FPG levels (all *p* < 0.05, Spearman correlation coefficient). 1-h PPG level of oGTT was negatively correlated only with glutamine, glycine and histidine levels (all *p* < 0.05, Spearman correlation coefficient).

BMI was negatively correlated with asparagine, glycine and ornithine levels (all *p* < 0.05, Spearman correlation coefficient). Total weight gain during pregnancy was positively correlated with alanine, histidine, tryptophan and tyrosine and negatively correlated with betaine and acetylcarnithine levels in the second trimester of pregnancy (all *p* < 0.05, Spearman correlation coefficient).

Regarding the FFAs, FPG level during oGTT in the second trimester of pregnancy was positively correlated with docosahexaenoic, oleic, palmitic, heptadecanoic and stearic acid levels, as well as the sums of SFAs, MUFAs and PUFAs and, also total sum of all analysed FFAs (all *p* < 0.05, Spearman correlation coefficient). 1-h PPG during oGTT was positively correlated with oleic acid levels, MUFAs and PUFAs (all *p* < 0.05, Spearman correlation coefficient). 2-h PPG during oGTT was positively correlated only with docosahexanoic acid levels (all *p* < 0.05, Spearman correlation coefficient). AUC under oGTT curve in the second trimester of pregnancy was positively correlated with docosahexanoic, oleic acid levels (all *p* < 0.05, Spearman correlation coefficient).

### 3.3. Association of AAs and FFAs Levels with Maternal and Neonatal Peripartal Data

As shown in Table 2, we found no statistically relevant differences in the occurrence of adverse peripartal outcomes neither between GDM vs. control group nor between post-GDM normal vs. PGI group. Glutamine levels were increased in the second trimester in women having macrosomic child (*p* = 0.032, Mann-Whitney), while alanine and tyrosine levels were decreased (*p* = 0.034 and *p* = 0.041 resp., Mann-Whitney). Palmitic, palmitooleic acid and choline levels were increased in GDM women who delivered by Caesarean section compared to women with GDM group who gave birth vaginally (*p* = 0.037, *p* = 0.016 and *p* = 0.012 resp., Mann-Whitney).

### 3.4. Comparison of AAs and FFAs Between GDM Sub-Groups Defined by the Presence or Absence of PGI

**Figure 2:**
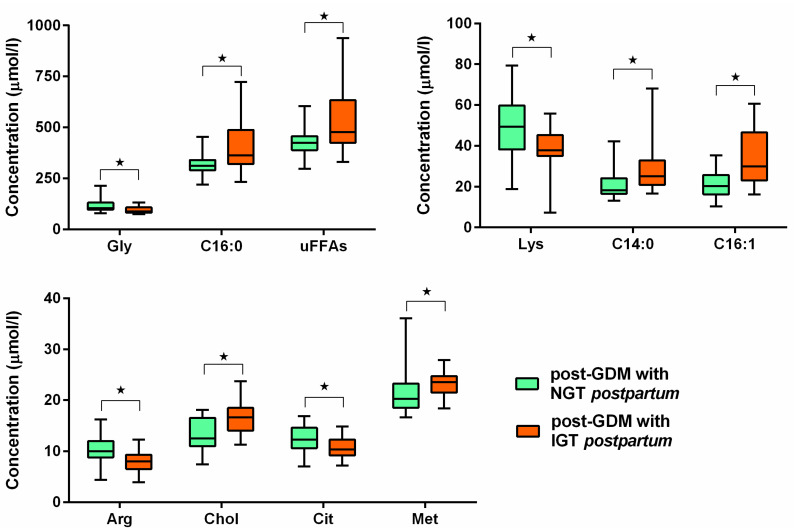
Comparison of AAs and FFAs levels during second trimester of pregnancy between GDM sub-groups defined by the presence or absence of PGI. ⋆ *p* < 0.05.

The PGI sub-group did not differ in age or pre-gestational BMI, however the prevalence of pre-gestational obesity was higher (*p* = 0.03, Fischer exact test), and FPG during pregnancy was higher (*p* = 0.007, Mann-Whitney), see Table 1, right side. Analysing this sub-group, levels of tetradecanoic, palmitic, palmitooleic acid and the sum of SFAs in mid-trimester were higher compared to post-GDM normalized group; also choline and methionine levels during the second trimester were higher. On the contrary, arginine, citrulline, glycine and lysine levels were lower (all *p* < 0.05, Mann-Whitney), for details see Figure 2.

In multivariate logistic regression analysis, we first compared the best predictors for GDM development. Naturally, FPG above 5.1 mmol/L was found as a most relevant predictor for PGI and therefore was not included in the following analyses. Three metabolites—increased docosahexaenoic acid and decreased cysteine and histidine levels and BMI were found the best the predictors of GDM development. AUC for ROC curve was 0.943 (Specificity 0.950, Sensitivity 0.912 for cut-off score 0.621), see Table 3 for details. Subsequently, we searched for predictors of postpartum PGI. Four metabolites (increased tetradecanoic acid and choline levels and decreased citrulline and glutamate levels) were found to predict postpartum PGI most strongly. AUC for ROC curve was 0.944 (Specificity 0.789, Sensitivity 0.929 for cut-off score 0.218), see Table 4 for details. Specific statistical analyses see in Supplementary Materials S1.

## 4. Discussion

In the present study, we determined plasma levels of selected AAs and FFAs in pregnant women with and without GDM and, additionally, analysed the same metabolites postpartum in women with normalised glucose tolerance and those with PGI. The panel of AAs and FFAs has been selected based on previously published data suggesting their predictive potential for development of T2DM [18,19] since GDM and T2DM share some metabolic features (mainly insulin resistance) and clinical phenotypes (components of metabolic syndrome). Major findings of the current study can be summarised as follows: (1) decreased levels of tetradecanoic acid and several AAs (alanine, asparagine, aspargic acid, citrulline, glutamine, glutamic acid, glycine, histidine, hydroxyproline, lysine, ornithine and serine) were found in GDM group during pregnancy compared to controls. Furthermore, (2) oGTT values in the pregnancy were positively correlated with oleic and docosahexaenoic acid, while almost all AAs were correlated negatively. Moreover, (3) certain differences in AA levels were detected in women with macrosomic babies. Finally, (4) logistic regression model (using AAs, FFAs and BMI) identified higher citrulline and glutamate levels and lower tetradecenoic acid and choline as the best predictors for postpartum PGI. So the hypothesis was accepted.

We found most AAs (12 of 20) decreased during mid-trimester of pregnancy in women with GDM compared to controls and most of them were subsequently normalised (i.e., increased) postpartum. Of FFAs analysed, only tetradecanoic acid was lower in GDM women in pregnancy. Statistically significant negative correlations of most of AAs levels with respective parameters of oGTT in the second trimester of pregnancy were detected with one exception being hydroxyprolin without statistically significant correlation. Lower asparagine, glycine and ornithine levels during pregnancy in GDM women could be explained by higher BMI in GDM women (negative correlation to BMI). Valine was found positively correlated to FPG in second trimester of oGTT. Logistic regression identified docosahexanoic acid cysteine, histidine and BMI as predictors of GDM itself.

The ability of certain AAs including branched chain and aromatic AAs (leucine, isoleucine, valine, tyrosine and phenylalanine) to predict T2DM development is well described. Studies reporting relationship between certain AAs and GDM have also been published. Metabolomic profiling of the amino acids may be useful in early diagnosis of GDM, however more data about their association with glucose homeostasis and hyperinsulinemia during pregnancy require clear evidence. Disturbances of AAs metabolism have already been demonstrated in GDM. Decreased levels of AAs during GDM complicated pregnancy were already found [10]. Similarly to our study Pappa et al. [10] examined fasting samples of women of the same ethnic group (Greek) at mid-pregnancy (30–33th week of gestation) with oGTT during 26th week of gestation. Other studies used either different diagnostic criteria or different sample collection period which can be crucial for results interpretation. Cetin et al. [7] used 100 g glucose load for GDM diagnosis analysed as 1-h, 2-h and 3-h PPG and maternal blood samples were obtained at the time of elective caesarean section. Rahimi et al. [8] analysed Iranian women with oGTT values based on ADA criteria, this study did not specify the period of sample collection. Bentley-Lewis [9] used third trimester oGTT for GDM diagnosis, but samples were provided during the first prenatal visit.

Lower levels of AAs during second trimester compared to their postpartum levels in GDM women may be caused by increased glucose and nutrient supply to the foetus, with exception of histidine and threonine levels which were higher during pregnancy. Interestingly choline levels were not higher in GDM group, but they were positively correlated do oGTT values and levels of several FFAs.

In our study total maternal plasma FFAs were positively associated with FPG, and other positive correlations could be a marker of the degree of carbohydrate disturbance during pregnancy, however only decreased levels of tetradecanoic acid were found in GDM women compared to controls. We have not found a difference between FFAs in GDM women which is probably caused by high interindividual variability of their levels. It is important to realize that maternal serum triacylglycerols did not cross placenta, only FFAs are able do so. Triacylglycerols have to be hydrolysed to FFAs by placental lipoprotein lipase at first. Increased levels of FFAs during pregnancy compared to their respective postpartum levels and positive correlations of respective parameters of oGTT with their levels probably reflect increased insulin resistance during second trimester. Positive correlations between branched-chain AAs and several FFAs could be one of the links between altered energy metabolisms during pregnancy. Branched chain AAs were already described to contribute to impaired glucose homeostasis and also their presence in plasma is associated with accumulation of long-chained FFAs.

A number of studies with different design and sample size measured FFA in pregnant women with GDM. A recent study compared FFA in women with GDM and healthy pregnant women in the blood samples collected at birth and in the cord blood [20]. Most of analysed FFA were lower in GDM group while AA and DHA did not differ between groups. Similarly, most FFA were lower in cord blood of women with GDM. In another study, lipid profile was determined in the group of 805 prospectively followed pregnant women of whom 8.3% (i.e., 67 women) developed GDM [21]. Women with GDM had higher triglycerides, lower HDL cholesterol and higher FFA. A prospective analysis of a large cohort of generally healthy women showed that certain FFAs might affect the risk of developing GDM probably through the negative association with insulin resistance (measured as HOMA-IR) and insulin secretion (measured as C-peptide) [22]. Certain FFAs were also associated (correlated) with plasma levels of cytokines and adipokines. Comprehensive metabolomics analysis of FFAs, AAs, bile acids and other metabolites was performed in women with GDM and in healthy control subjects in the pregnancy [23]. Using ROC analysis, the authors computed diagnostic model for GDM containing BMI, RBP4, n-acetyl-aspartic acid and palmitoleic acid (C16:1). Lipid metabolism parameters were investigated in the smaller cohort of women—29 had GDM and 33 were healthy pregnant controls [24]. Changes in lipid profiles were found in women with GDM. Furthermore, the amount of GPR120, the receptor for FFA, was higher in peripheral blood monocytes in GDM group with potential effect on the control of lipid metabolism through FGF21.

Different approach was used in another study where the authors measured SFA repeatedly throughout pregnancy (4-times) [25]. They found that certain SFA (e.g., palmitic acid) were significantly higher already in 10–14th gestational week and increased the risk of GDM. Fatty acid profiles of erythrocyte membranes were investigated in 32 women with GDM and 11 healthy pregnant controls [26]. Several FA were expressed differentially between the groups, 3 saturated (myristic, palmitic and stearic) were lower in GDM group. Plasma phospholipid fatty acids did not differ between GDM and control subjects in the pregnancy and postpartum (before and after delivery) [27]. However, desaturase activity (deduced from precursor to product ratio of FA) was increased. Finally, a systematic meta-analysis focused on plasma levels of FFAs in women with GDM and healthy pregnant controls [28]. Women with GDM had higher levels of FFAs.

Our study found increased glutamine, alanine and tyrosine levels decreased in second trimester in those women, who had macrosomic child. Maternal insulin is a primary regulator of amino acid concentrations. Maternal plasma amino acid concentrations are a major determinant of fetal growth, because they provide essential substrate for this process, including branched-chain amino acids. An active transport system for amino acids exists in the placenta that promotes a higher fetal-maternal plasma concentration ratio. This ratio remains fairly constant throughout mid- and late gestation with a good proportional correlation between specific amino acids in maternal and fetal plasma. Total a-amino acid nitrogen levels in maternal blood relate significantly to birth weight in normal pregnancies [29].

The effect of GDM pregnancies may affect child development, thus increased foetal fat mass together with foetal hyperinsulinemia may be the result of GDM pregnancy, however, this is not simply related to maternal hyperglycaemia and plasma AAs together with FFAs levels could provide clear evidence of excessive nutrient supply to the foetus.

In our study 14 of 34 GDM women had any form of persisted glucose intolerance after delivery. However, this proportion doesn’t reflect the true incidence of PGI after delivery since we ascertain the incidence in a previous larger study around 17% in Czech population [30] and here we included only a subset of women from a wider study [31]. In those GDM patients who developed PGI—citrulline and glycine levels were even lower in mid-trimester of pregnancy than those found in GDM compared to controls. On the other hand, levels of several FFAs (C14:0, C16:0 and C16:1) and the sum of analysed SFAs were higher. Similarly, methionine and choline levels, which is not surprising because choline levels were positively associated with oGTT values. Lower arginine levels were also described. The question remains if they could be used as a possible predictor of diabetes development in early period after delivery, moreover, the dynamics of AAs and FFAs levels during pregnancy definitely requires further study.

A big role can play diet and a gut microbiome in FFAs and AAs levels. Alterations in the gut microbiome are significantly associated with levels of circulating linoleic acid [32], indicating that some of the significant changes in FFAs and AAs may be significantly linked to the gut microbiome and its products, including the short-chain fatty acid, butyrate [33]. However the connection to GDM has not been studied yet.

The limitation of the study is definitely the number of participants, however, the study was designed as a pilot study and we are going to repeat the measurement on the larger set of people. Unfortunately, we did have neither pre-pregnant data nor data of healthy pregnant women after delivery, so we cannot answer the possible question, if there is any shift in FFAs or AAs levels in healthy women during pregnancy.

## 5. Conclusions

In this pilot study we measured plasma levels of FFAs and AAs during pregnancy in women with normal pregnancy and GDM and, also in post-GDM women with PGI after delivery. Our findings support a possible link between GDM development and PGI after delivery and selected metabolite levels. The predictive potential of plasma FFAs and AAs levels on a diabetes risk in future life requires further validation.

## Figures and Tables

**Table 1:** Clinical and anthropometric characteristics of study subjects.

Parameter	GDM (*n* = 34)	Controls (*n* = 20)	*p*	GDM Normalized After Delivery (*n* = 20)	GDM with Persisting PGI (*n* = 14)	*p*
Age (years)	33 [30–35]	32 [29.5–35]	NS	33 [31–36]	31.5 [30–34]	NS
Pre-gestational BMI (kg/m^2^)	25.6 [21.7–28.4]	21.3 [20.1–23.5]	0.007	26.5 [21.8–27.6]	24 [20.4–30.6]	NS
Obesity (pre-gest. BMI > 30 kg/m^2^)	17.6%	0%	NS	5.0%	35.7%	0.03
Weight gain during pregnancy (kg)	6 [4–10]	13 [10–17]	0.005	6 [4–9]	9 [6–11]	NS
Offspring birth weight (g)	3300 [3050–3550]	3020 [2900–3450]	NS	3200 [3020–3500]	3360 [3060–3630]	NS
History of previous GDM (number of nulliparous)	23.5% (14)	5.0% (10)	NS	15.0% (8)	42.8% (6)	NS
Family history of DM	76.5%	40.0%	0.01	70.0%	92.9%	NS
FPG (mmol/L) mid-gestation	4.9 [4.6–5.3]	4.0 [3.8–4.3]	<1 × 10^−6^	4.6 [4.4–5.0]	5.3 [4.9–6.0]	0.007
1-h PPG (mmol/L) mid-gestation	9.2 [8.8–10.1]	5.8 [5.1–7.6]	1 × 10^−6^	9.7 [9.1–10.1]	8.3 [8.2–6.0]	NS
2-h PPG (mmol/L) mid-gestation	8.0 [7.7–8.6]	4.8 [4.2–6.6]	<1 × 10^−6^	8.1 [7.6–8.6]	8.0 [7.8–8.8]	NS
AUC_oGTT_ (mmol/L/h) mid-gestation	13.0 [12.5–13.3]	8.9 [8.2–10.6]	<1 × 10^−6^	12.8 [12.3–13.2]	13.1 [12.8–14.6]	NS
Insulin treatment	44.0%	-	-	35.0%	50.0%	NS

Data expressed as medians and [IQR] or proportions. Differences were evaluated by nonparametric Mann-Whitney or Fischer exact tests. 1-h PPG—1-h post-load plasma glucose, 2-h PPG—2-h post-load plasma glucose, AUC_oGTT_—area under the oGTT curve, oGTT—oral glucose tolerance test, BMI—body mass index, DM—diabetes mellitus, FPG—fasting plasma glucose, GDM—gestational diabetes mellitus, NS—not statistically significant; PGI—postpartum glucose intolerance.

**Table 2:** Comparison of peripartal adverse outcomes in women with and without GDM, resp. with and without PGI.

Parameter (*n*/%)	GDM (*n* = 34)	Controls (*n* = 20)	*p*	GDM Normalized After Delivery (*n* = 20)	GDM with Persisting PGI (*n* = 14)	*p*
Macrosomia (birthweight > 4000 g)	3/8.8%	1/5.0%	NS	1/5.0%	2/14.2%	NS
Pre-term delivery (<38th week of gestation)	2/5.9%	2/10.0%	NS	1/5.0%	1/7.1%	NS
Delivery induction (oxytocin or Prostaglandin E)	8/23%	1/5.0%	NS	7/35%	1/7.1%	NS
Non-physiologic delivery (Caesarean section, VEX, forceps)	9/26.5%	4/20%	NS	3/15%	6/42.9%	NS
Prolonged delivery (>480 min)	1/2.9%	0/0%	NS	0/0%	1/7.1%	NS
Delivery complications (manual extraction of placenta, hypotonia of uterus)	1/2.9%	2/10.0%	NS	0/0%	1/7.1%	NS
Abnormal Apgar score in 5th min (<5)	0/0%	0/0%	NS	0/0%	0/0%	NS
Abnormal cord blood pH (<7.1)	0/0%	0/0%	NS	0/0%	0/0%	NS
Abnormal BE (<−12)	0/0%	0/0%	NS	0/0%	0/0%	NS
Any of the peripartal adverse outcomes (any of those above)	17/50%	8/40%	NS	9/45%	8/57.1%	NS
Any of the offspring’s adverse outcome *	3/8.8%	1/5.0%	NS	1/5.0%	2/14.2%	NS

Comparisons were performed using chi-square test. GDM—gestational diabetes mellitus; PGI—postpartum glucose intolerance; NS—not statistically significant; VEX—vacuum extractor; BE—base excess. * One or more of the following outcomes: pathological Apgar score, cord blood pH, BE, macrosomia.

**Table 3:** Logistic regression model, best predictive markers for GDM prediction.

Predictive Markers for GDM Prediction	Model Coeficients	OR	95% CI for OR	*p*-Values
BMI	0.53	1.70	(1.15–2.51)	0.007
docosahexaenoic acid	0.11	1.12	(1.03–1.21)	0.009
cystein	−0.57	0.57	(0.35–0.91)	0.019
histidine	−0.24	0.78	(0.67–0.92)	0.003

**Table 4:** Logistic regression model, best predictive markers for PGI prediction.

Predictive Markers for PGI Prediction	Model Coeficients	OR	95% CI for OR	*p*-Values
choline	0.78	2.19	(1.01–4.76)	0.048
tetradecanoic acid	0.20	1.23	(0.97–1.54)	0.085
glutamate	−0.26	0.77	(0.60–0.99)	0.041
citruline	−0.43	0.65	(0.36–1.19)	0.165

## Data Availability

Data are available with authors.

## Supplementary Materials

The following supporting information can be downloaded at: https://www.mdpi.com/article/10.3390/jcm14134744/s1, Table S1: Models for Table 3 and Table 4 and ROC curves and AUC for final model.

## Abbreviations

1-h PPG	1-h post-load plasma glucose
2-h PPG	2-h post-load plasma glucose
AAs	amino acids
ADA	American Diabetes Association
AUC_oGGT_	area under the oGTT curve
BE	base excess
BMI	body mass index
CI	confidence interval
DIP	diabetes in pregnancy
DM	diabetes mellitus
FFAs	free fatty acids
FPG	fasting plasma glucose
GDM	gestational diabetes mellitus
IADPSG	International Association of Diabetes in Pregnancy Study Group
IQR	interquartile range
MUFAs	monosaturated fatty acids
oGTT	oral glucose tolerance test
OR	odds ratio
PGI	postpartum glucose intolerance
PUFAs	polysaturated fatty acids
ROC	Receiver Operating Characteristic
SFAs	saturated fatty acids
T2DM	type 2 diabetes mellitus
VEX	vacuum extractor
WHO	World Health Organization
